# Megaloblastic anemia-related iron overload and erythroid regulators: a case report

**DOI:** 10.1186/s13256-021-03065-0

**Published:** 2021-09-20

**Authors:** Nicolas Vallet, Jean-Baptiste Delaye, Martine Ropert, Amélie Foucault, Noémie Ravalet, Sophie Deriaz, Thomas Chalopin, Hélène Blasco, François Maillot, Olivier Hérault, Emmanuel Gyan

**Affiliations:** 1 Department of Hematology and Cell Therapy, Tours University Hospital, 2 Boulevard Tonnellé, 37044 Tours Cedex, France; 2 Department of Biochemistry and Molecular Biology, Tours University Hospital, Tours, France; 3 Department of Biochemistry, CHU Rennes, Rennes, France; 4 Department of Biological Hematology, Tours University Hospital, Tours, France; 5 CNRS ERL7001 LNOX, EA 3549, Tours University, Tours, France; 6 Department of Internal Medicine, Tours University Hospital, Tours, France

**Keywords:** Vitamin B12, Iron overload, Erythropoiesis, Erythroferrone, GDF15, Case report

## Abstract

**Background:**

In ineffective erythropoiesis, hepcidin synthesis is suppressed by erythroid regulators, namely erythroferrone and growth differentiation factor-15. For the first time, the hypothesis that iron overload in megaloblastic anemia may be related to ineffective erythropoiesis is explored by describing the kinetics of hepcidin, erythroferrone, and growth differentiation factor-15 levels in a patient diagnosed with megaloblastic anemia associated with iron overload.

**Case presentation:**

An 81-year-old Caucasian male was admitted for fatigue. He had type-2 diabetes previously treated with metformin, ischemic cardiac insufficiency, and stage-3 chronic kidney disease. Vitiligo was observed on both hands. Biological tests revealed normocytic non-regenerative anemia associated with hemolysis, thrombocytopenia, and elevated sideremia, ferritin, and transferrin saturation levels. Megaloblastic anemia was confirmed with undetectable blood vitamin B12 and typical cytological findings like hyper-segmented neutrophils in blood and megaloblasts in bone marrow. The patient received vitamin B12 supplementation. At 3 months, biological parameters reached normal values. Hepcidin kinetics from diagnosis to 3 months inversely correlated with those of erythroferrone and growth differentiation factor-15.

**Conclusions:**

This case suggests that iron-overload mechanisms of dyserythropoietic anemias may apply to megaloblastic anemias.

## Background

Iron overload related to hemolysis and ineffective erythropoiesis is a feature of megaloblastic anemia. The latter mechanism is yet to be explored [1, 2]. In ineffective erythropoiesis, hepcidin synthesis is suppressed by erythroid regulators, namely erythroferrone (ERFE) [3] and growth differentiation factor-15 (GDF15) [4]. Hepcidin is essential for iron homeostasis, as it induces the endocytosis of ferroportin, lowering iron absorption and circulating iron levels through iron sequestration by hepatocytes, macrophages, and duodenal enterocytes [5]. Herein, we describe the kinetics of hepcidin, ERFE, and GDF15 levels in a patient diagnosed with megaloblastic anemia associated with iron overload. We hypothesized that ineffective erythropoiesis was associated with high levels of ERFE and GDF15 at the time of diagnosis, possibly associated with suppressed hepcidin, thus explaining the iron overload.

## Case presentation

**Fig. 1:**
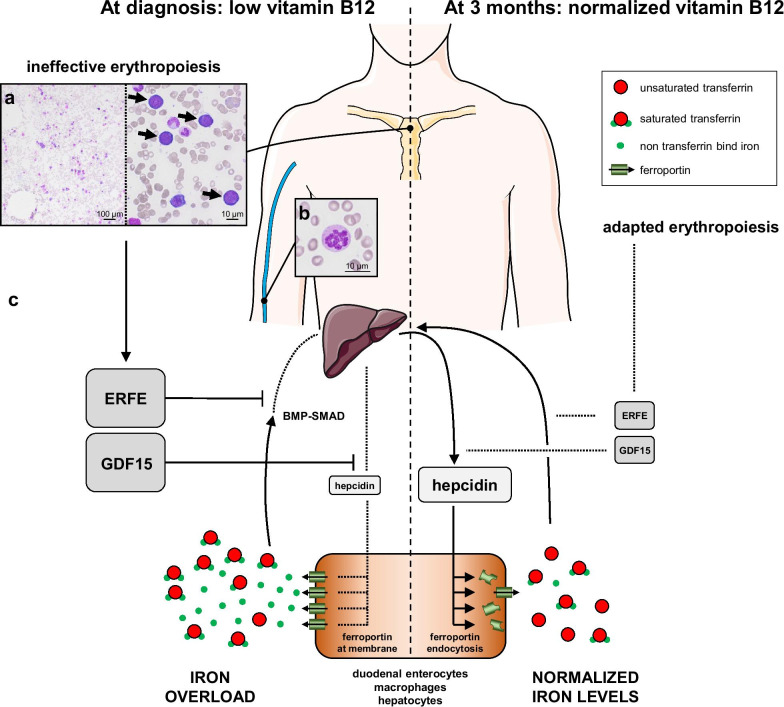
Proposed integrative interpretation of the results obtained from this case report.** a** May–Grünwald Giemsa staining of a bone-marrow smear showing hypercellularity (left) and intense basophilic megaloblasts (right, arrows). **b** Peripheral blood smear revealing increased neutrophil cell volume with a hypersegmented nucleus. **c** Hypothetical mechanism of the pathogenesis of iron overload in megaloblastic anemia. Relative changes in the size of the protein names represent their down- or upregulation. *ERFE* erythroferrone, *GDF15* growth differentiation factor-15

An 81-year-old Caucasian male was referred to emergency department for fatigue associated with profound anemia. He had type-2 diabetes previously treated with metformin, ischemic cardiac insufficiency, and stage-3 chronic kidney disease (CKD, CKD-Epidemiology Collaboration [CKD-EPI] estimated glomerular filtration rate: 32 mL/minute/1.73 m^2^). There were no clinical signs of infectious or tumoral disease. Vitiligo was observed on both hands. First biological tests revealed normocytic non-regenerative anemia associated with hemolysis, thrombocytopenia, and elevated sideremia, ferritin, and transferrin saturation (TSAT) levels. Vitamin B12 was undetectable, whereas vitamin B9 and C-reactive protein levels were normal (Table 1). He received one pack of red blood cells (RBC), then was admitted to internal medicine department. A peripheral blood smear showed hypersegmented neutrophils, and a bone-marrow smear showed hypercellularity and clear signs of dyserythropoiesis and blocked maturation of erythroid cells (Fig. 1a, b). Stomach endoscopy and biopsies showed chronic gastritis and fundic atrophy. Though metformin may have participated in this condition [1], a diagnosis of megaloblastic anemia caused by pernicious anemia was preferred considering the profound anemia and vitiligo, even if blood antiparietal cells or anti-intrinsic-factor antibodies were not detected. After two other packs of RBC and five daily intramuscular vitamin B12 injections, he was discharged and continued oral vitamin B12 supplementation. Three-month (M3) biological control revealed that platelet count and hemoglobin, sideremia, and TSAT levels normalized (Table 1). Ferritin returned to normal values at 7 months (361 µg/L).

**Table 1:** Peripheral blood characteristics at diagnosis and 3 months after vitamin B12 supplementation

Biological parameters	Unit	At diagnosis	3 months later	Laboratory reference range
Hemoglobin	g/L	57	129	129–170
Mean corpuscular volume	fL	99	90	80–100
Reticulocytes	10^9^/L	16	94	NA
Absolute neutrophil count	10^9^/L	2.2	5.4	1.5–7.5
Platelets	10^9^/L	89	240	150–400
Creatinine	µmol/L	171	200	59–104
Estimated glomerular filtration rate (CKD-EPI formula)	mL/minute/1.73 m^2^	32	26	NA
Total bilirubin	µmol/L	31	9	2–24
Indirect bilirubin	µmol/L	20	5	2–17
Haptoglobin	g/L	0.4	1.8	0.5–2.0
Lactate dehydrogenase	U/L	910	164	135–225
Sideremia	µmol/L	36	13	5.8–34.5
Ferritin	µg/L	537	437	30–400
Transferrin	g/L	1.60	2.10	2.0–3.6
Transferrin saturation coefficient	%	88	25	25–40
Reactive C protein	mg/L	1.2	0.9	0.3–5.0
Vitamin B9	nmol/L	21	–	8.8–60.8
Vitamin B12	pmol/L	<111	743	145–569
Erythropoietin	mUI/mL	43.9	8.6	2.6–18.5
GDF15	pg/mL	> 8000	2,240	1080–3700*
Erythroferrone	ng/mL	14.44	0.53	0.01–1.92*
Hepcidin^†^	nmol/L	10.6	28.7	4–30

CKD-EPI indicates chronic Kidney Disease Epidemiology Collaboration. *Reference range was defined with healthy controls matched for age. ^†^To convert hepcidin from nmol/L to ng/mL, multiply by 2.76

We tested our hypothesis by analyzing the samples at the time of diagnosis and at M3 after receiving informed consent of the patient. Serum hepcidin, ERFE, and GDF15 levels were assessed by ELISA (hepcidin-25: S-1337, Peninsula; ERFE: ERF-001, Intrinsic LifeSciences; GDF15: DGD150, R&D Systems). Serum erythropoietin (EPO) was measured by chemiluminescence (UniCel DxL, Beckman Coulter).

ERFE and GDF15 levels were higher than those of age-matched healthy controls (HC) at diagnosis, dropping to HC levels at M3, supporting our hypothesis. The level of hepcidin was lower at the time of diagnosis than M3 but remained within reference levels, and its kinetics from diagnosis to M3 inversely correlated with those of EPO, ERFE, and GDF15 (Table 1).

## Discussion and conclusions

Given the high level of TSAT, the level of hepcidin at diagnosis was inappropriately low, suggesting suppression by an erythroid regulator. The influence of CKD [6, 7] and metformin [8] may explain why the hepcidin levels were comparable to those of HC, but its range is consistent with that found in CKD patients [7]. A metabolic syndrome may also explain high ferritin levels. But these potential biases were internally controlled by comparing two time points before and after vitamin B12 and hemoglobin normalization, when comorbidities and treatments were comparable. ERFE and GDF15 levels were higher than in HC from our institution and previous studies [9, 10]. ERFE levels at diagnosis were comparable to those of patients with pyruvate-kinase deficiency but lower than those with β-thalassemia [9]. The inverse correlation of EPO and ERFE kinetics is consistent with that observed in EPO-treated healthy humans [3] and CKD mouse [6] models. The GDF15 level at diagnosis was comparable to that of congenital dyserythropoietic anemia type-1 [11]. Despite the undetectable vitamin B12 levels and typical cytological abnormalities that define megaloblastic anemia, the mean corpuscular volume (MCV) was unexpectedly not macrocytic [1]. The patient had no history of iron deficiency, inflammation, or red-blood-cell transfusion. GDF15 was recently shown to stimulate erythroid precursor growth in mice [12]. Cell-cycle acceleration in erythroid precursors may have hidden macrocytosis, but whether high levels of GDF15 may have affected the MCV remains to be demonstrated. Intramedullary hemolysis, suggested by bilirubin, lactate dehydrogenase, and haptoglobin levels, can also explain iron overload [2]. However, hemolysis alone cannot explain the higher ERFE and GDF15 levels. In sickle-cell diseases (SCD), in which hemolysis is dominant compared with dyserythropoiesis, the ERFE and GDF15 levels and their correlation with hepcidin levels were not comparable to those observed in β-thalassemia [13]. Additionally, the hepcidin level was inappropriately low relative to that at M3 when TSAT was normal, reinforcing the hypothesis that an erythroid regulator suppressed hepcidin synthesis.

ERFE suppresses hepcidin synthesis by sequestering bone morphogenetic protein (BMP) receptor ligands [5]. Its synthesis starts upon EPO stimulation through the JAK2-STAT5 pathway in erythroblasts [14]. ERFE levels are elevated in human congenital or acquired dyserythropoietic diseases associated with iron overload [3, 15]. GDF15 was shown to suppress hepcidin synthesis in the context of β-thalassemia [4] and congenital dyserythropoietic anemia [11]. GDF15 is produced by erythroblasts, but its mechanisms of synthesis and hepcidin suppression are not fully understood [4, 10].

In conclusion, we found the kinetics of hepcidin, ERFE, and GDF15 to be comparable to those described in iron overload associated with ineffective erythropoiesis (Fig. 1c). This case broadens the spectrum of iron-overload mechanisms in dyserythropoietic anemias to vitamin B12 deficiency, in which hepcidin levels inversely correlate with those of erythroid regulators, suggesting suppression by ERFE and GDF15.

## Data Availability

The dataset analyzed during the current study is available from the corresponding author on reasonable request.

## Abbreviations

BMP: Bone morphogenetic protein
CKD: Chronic kidney disease
EPO: Erythropoietin
ERFE: Erythroferrone
GDF15: Growth differentiation factor-15
M3: Three months
MCV: Mean corpuscular volume
RBC: Red blood cells
SCD: Sickle cell disease
TSAT: Transferrin saturation

## References

[1] Green R. Vitamin B12 deficiency from the perspective of a practicing hematologist. Blood. 2017;129(19):2603–11. 10.1182/blood-2016-10-569186 28360040

[2] Ho C-H, You J-Y, Chau W-K,* et al*. Diagnostic value of serum transferrin receptor and glycosylated hemoglobin on hemolytic anemia. Ann Hematol. 2003;82(4):228–30. 10.1007/s00277-003-0623-3 12707725

[3] Ganz T, Jung G, Naeim A,* et al*. Immunoassay for human serum erythroferrone. Blood. 2017;130(10):1243–6. 10.1182/blood-2017-04-777987 PMC5606005 28739636

[4] Tanno T, Bhanu NV, Oneal PA,* et al*. High levels of GDF15 in thalassemia suppress expression of the iron regulatory protein hepcidin. Nat Med. 2007;13(9):1096–101. 10.1038/nm1629 17721544

[5] Camaschella C, Nai A, Silvestri L. Iron metabolism and iron disorders revisited in the hepcidin era. Haematologica. 2020;105(2):260–72. 10.3324/haematol.2019.232124 PMC7012465 31949017

[6] Hanudel MR, Rappaport M, Chua K,* et al*. Levels of the erythropoietin-responsive hormone erythroferrone in mice and humans with chronic kidney disease. Haematologica. 2018;103(4):e141–2. 10.3324/haematol.2017.181743 PMC5865435 29419424

[7] Coll AP, Chen M, Taskar P,* et al*. GDF15 mediates the effects of metformin on body weight and energy balance. Nature. 2020;578(7795):444–8. 10.1038/s41586-019-1911-y PMC7234839 31875646

[8] Ashby DR, Gale DP, Busbridge M,* et al*. Plasma hepcidin levels are elevated but responsive to erythropoietin therapy in renal disease. Kidney Int. 2009;75(9):976–81. 10.1038/ki.2009.21 19212416

[9] van Vuren AJ, Eisenga MF, van Straaten S,* et al*. Interplay of erythropoietin, fibroblast growth factor 23, and erythroferrone in patients with hereditary hemolytic anemia. Blood Adv. 2020;4(8):1678–82. 10.1182/bloodadvances.2020001595 PMC7189297 32324886

[10] Theurl I, Finkenstedt A, Schroll A,* et al*. Growth differentiation factor 15 in anaemia of chronic disease, iron deficiency anaemia and mixed type anaemia. Br J Haematol. 2010;148(3):449–55. 10.1111/j.1365-2141.2009.07961.x 19863534

[11] Casanovas G, Swinkels DW, Altamura S,* et al*. Growth differentiation factor 15 in patients with congenital dyserythropoietic anaemia (CDA) type II. J Mol Med. 2011;89(8):811–6. 10.1007/s00109-011-0751-5 21475976

[12] Hao S, Xiang J, Wu D-C,* et al*. Gdf15 regulates murine stress erythroid progenitor proliferation and the development of the stress erythropoiesis niche. Blood Adv. 2019;3(14):2205–17. 10.1182/bloodadvances.2019000375 PMC6650738 31324641

[13] Mangaonkar AA, Thawer F, Son J,* et al*. Regulation of iron homeostasis through the erythroferrone-hepcidin axis in sickle cell disease. Br J Haematol. 2020;189(6):1204–9. 10.1111/bjh.16498 PMC8011855 32030737

[14] Kautz L, Jung G, Valore EV, Rivella S, Nemeth E, Ganz T. Identification of erythroferrone as an erythroid regulator of iron metabolism. Nat Genet. 2014;46(7):678–84. 10.1038/ng.2996 PMC4104984 24880340

[15] Bondu S, Alary A-S, Lefèvre C,* et al*. A variant erythroferrone disrupts iron homeostasis in SF3B1-mutated myelodysplastic syndrome. Sci Transl Med. 2019. 10.1126/scitranslmed.aav5467. PMC8005358 31292266

